# Neonatal bisphenol A exposure induces meiotic arrest and apoptosis of spermatogenic cells

**DOI:** 10.18632/oncotarget.7218

**Published:** 2016-02-06

**Authors:** Meina Xie, Pengli Bu, Fengjie Li, Shijian Lan, Hongjuan Wu, Lu Yuan, Ying Wang

**Affiliations:** ^1^ Medicine Experiment Center, Weifang Medical University, Wei Fang 261053, P. R. China; ^2^ School of Bioscience and Technology, Weifang Medical University, Wei Fang 261053, P. R. China; ^3^ Department of Biological Sciences, St. John's University, Queens, NY 11439, USA; ^4^ Department of Pharmaceutical Sciences, St. John's University, Queens, NY 11439, USA; ^5^ School of Basic Medical Sciences, Weifang Medical University, Wei Fang 261053, P. R. China

**Keywords:** bisphenol A, spermatogenic cells, meiotic arrest, Boule, estrogen receptor α/β

## Abstract

Bisphenol A (BPA) is a widely used industrial plasticizer, which is ubiquitously present in the environment and organisms. As an endocrine disruptor, BPA has caused significant concerns regarding its interference with reproductive function. However, little is known about the impact of BPA exposure on early testicular development. The aim of the present study was to investigate the influence of neonatal BPA exposure on the first wave of spermatogenesis. Newborn male mice were subcutaneously injected with BPA (0.01, 0.1 and 5 mg/kg body weight) daily from postnatal day (PND) 1 to 21. Histological analysis of testes at PND 22 revealed that BPA-treated testes contained mostly spermatogonia and spermatocytes with markedly less round spermatids, indicating signs of meiotic arrest. Terminal dUTP nick-end labeling (TUNEL) assay showed that BPA treatment significantly increased the number of apoptotic germ cells per tubule, which corroborated the observation of meiotic arrest. In addition, BPA caused abnormal proliferation of germ cells as revealed by Proliferating Cell Nuclear Antigen (PCNA) immunohistochemical staining. Mechanistically, BPA-treated testes displayed a complete lack of *BOULE* expression, which is a conserved key regulator for spermatogenesis. Moreover, BPA significantly increased the expression of estrogen receptor (ER) α and β in the developing testis. The present study demonstrated that neonatal BPA exposure disrupted meiosis progression during the first wave of spermatogenesis, which may be, at least in part, due to inhibition of *BOULE* expression and/or up-regulation of ERα/β expression in BPA-exposed developing testis.

## INTRODUCTION

Bisphenol A (BPA), an endocrine disrupting chemical, is widely used in the manufacture of a variety of domestic products including plastic food and water containers, food packaging, water pipes, baby bottles and toys (http://www.bisphenol-a.org). By 2003, over 6 billion pounds of BPA were generated in the manufacture processes [1]. Consequently, BPA has been detected in food, water, human urine and blood samples, placenta and amniotic fluid, as well as breast milk [2–6].

Exposure to BPA during early developmental stage was speculated to affect testicular development and spermatogenesis [7–10]. Some reports documented that ICR (Institute of Cancer Research) mice implanted with a tube filled with BPA (BPA was released at 60 μg/day) throughout pregnancy and lactation significantly lowered the percentage of seminiferous tubules with elongated spermatids in the testis of male offspring at age of 4 weeks [11]. Neonatal administration of a low dose (10 μg/kg) of BPA in both mouse and rat was demonstrated to cause deforming of the acrosomal granule and nucleus in step 2–3 spermatids [12]. However, there is still a need for studies, which assess the influence of BPA on spermatogenesis and testis development utilizing a dose range that spans from microgram to milligram per kilogram body weight. Particularly, a dose of 10 microgram per kilogram body weight is necessary for a neonatal exposure model as it correlates with human infant environmental exposure to BPA [13].

Recently, BPA exposure at an environmentally relevant dose was shown to induce meiotic abnormalities in adult male rats [14]. But the underlying mechanisms remain unknown. *Boule*, a member of the DAZ (Deleted in AZoospermia) family of genes, was reported to exhibit a male predominant expression pattern and to be required for spermatogenesis. In *Drosophila*, male *Boule* mutants are sterile, and their germ cells are arrested at the spermatocyte stage [15]. *Boule* homozygous mutant mice are also sterile with meiotic arrest at the round spermatid stage [16]. The lack of *BOULE* expression has also been associated with meiotic arrest during spermatogenesis in infertile male patients [17, 18]. Together, multiple lines of evidence indicate a conserved function of *Boule* in regulating spermatogenesis across species.

Moreover, a number of studies utilizing *in vivo* and/or *in vitro* systems showed that BPA interacted with estrogen receptor (ER) α and ERβ [19–21]. Izzotti A *et al.* found that BPA up-regulated ERβ in mouse mammary tissue [20]. Tabuchi *et al.* reported that BPA down-regulated ERα in an *in vitro* Sertoli cell model [19]. BPA has been shown to exert dual actions as either agonist or antagonist for ERα and/or ERβ in a cell type- and/or tissue type-specific manner [19, 22, 23]. Furthermore, the mode of action of BPA may also depend on the ER subtypes [24].

In the present study, we chose a high dose of BPA (5 mg/kg) equal to the No Observed Adverse Effect Level (NOAEL) (FAO/WHO 2008), a middle dose (0.1 mg/kg) and a low dose (0.01 mg/kg) below the tolerable daily intake levels of BPA (0.05 mg/kg) announced by European Food Safety Authority (EFSA, 2006) and the US Environmental Protection Agency (EPA, 1993). In the present report, male neonatal mice were injected subcutaneously from postnatal day (PND) 1 to 21 with BPA (0.01, 0.1 and 5 mg/kg), and the testes were harvested at PND 22 for investigation of the effects of neonatal BPA exposure on the first wave of spermatogenesis. Within these testes levels of apoptosis and proliferation were assessed. Testicular expression of Proliferating Cell Nuclear Antigen (PCNA), ERα, ERβ, and *BOULE* were examined to elucidate the potential mechanisms underlying the deleterious effects of BPA on spermatogenesis.

## RESULTS

### Testis histology was damaged by BPA exposure

**Figure 1:**
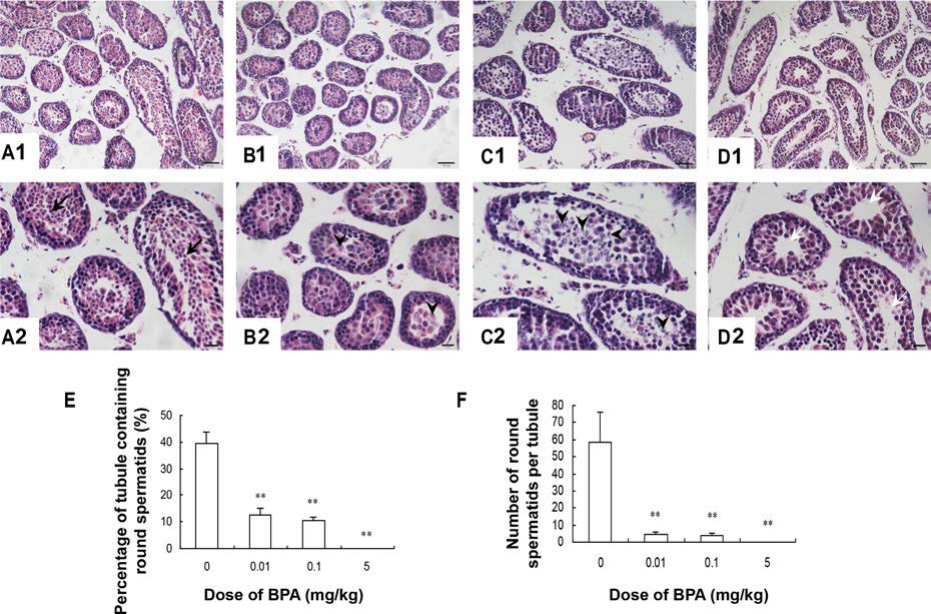
Testis histology was damaged by BPA exposure (**A**, **B**, **C**, **D**) are representative images of testis sections from control (coin oil), BPA (0.01 mg/kg), BPA (0.1 mg/kg) and BPA (5 mg/kg) groups. (**E**) Percentage of tubule containing round spermatids; (**F**) Number of round spermatids per tubule. Round spermatids (black arrows) were found in seminiferous tubules in control group (**A1**, **A2**). Degenerative spermatocytes (arrowhead) appeared in the centers of seminiferous tubules (**B1**, **B2**, **C1** and **C2**) in BPA-treated groups. Seminiferous tubule lumen appeared vacant (white arrows) in high-dose group (**D1**, **D2**). Scale bar (in **A1**, **B1**, **C1**, **D1**) = 50 μm; Scale bar (in **A2**, **B2**, **C2**, **D2**) = 20 μm. **p < 0.01 compared with control.

Histological examination revealed that the structure of the seminiferous tubule was damaged by BPA exposure during early development. In the vehicle-treated group, germ cells (including spermatogonia, spermatocytes and round spermatids) in seminiferous tubules were well-organized with abundant cytoplasm and nuclei (Figure 1A1, 1A2). However, in BPA-treated groups, very few round spermatids were found in the seminiferous tubules, indicating spermatogenic arrest at the spermatocyte level (*p* < 0.01, Figure 1B–1F). Furthermore, we found significantly more desquamated cells and chromosome fragments in the center of seminiferous tubules in BPA-treated groups (Figure 1B–1C). Interestingly, most desquamated cells were seen in the 0.1 mg/kg group (Figure 1C1, 1C2). At the highest dosing level, seminiferous tubule lumen appeared vacant, indicating a significant loss of developing germ cells (Figure 1D1, 1D2).

### BPA exposure induced germ cell apoptosis

**Figure 2:**
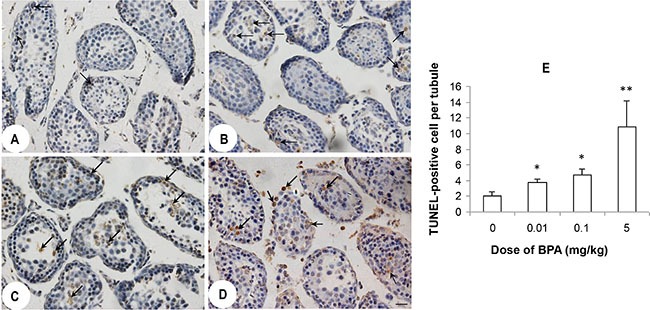
BPA exposure induced germ cell apoptosis (**A**, **B**, **C**, **D**) are representative TUNEL staining images of testis sections from control (coin oil), BPA (0.01 mg/kg), BPA (0.1 mg/kg) and BPA (5 mg/kg) groups. More TUNEL positive staining cells (black arrows) were found in BPA groups than control group. (**E**) Quantification of TUNEL positive staining in the testes from above groups. Shown is the number of TUNEL positive cells per seminiferous tubule from 100 tubule cross-sections from each mouse (*n* = 6 mice per group). *p < 0.05, **p < 0.01 compared with control.

BPA exposure increased the number of Terminal dUTP nick-end labeling (TUNEL) - positive germ cells in all dose groups (Figure 2B−2D) compared to control group (Figure 2A). Average number of TUNEL-positive cell per tubule significantly increased in BPA-exposed groups (*p* < 0.05, *p* < 0.01, Figure 2E). In addition, mostly desquamated cells in the centers of the seminiferous tubules were TUNEL-positive (Figure 2B–2D).

### BPA exposure caused abnormal proliferation of spermatogenic cells

**Figure 3:**
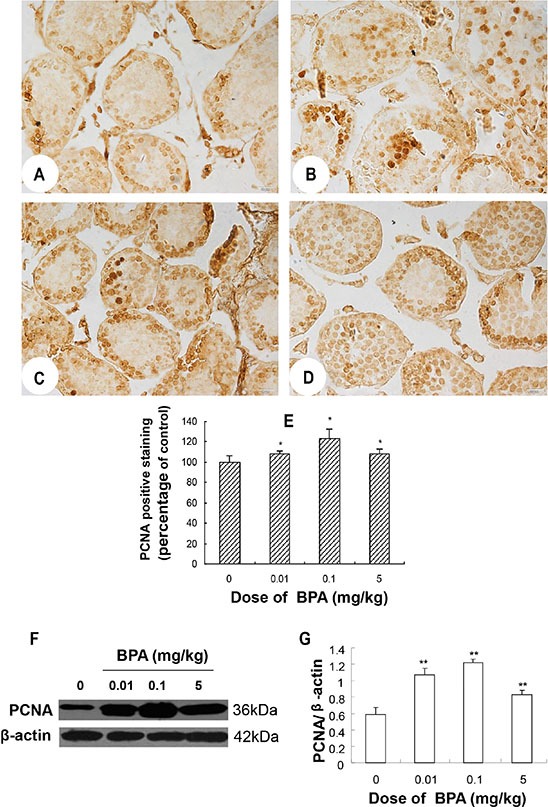
BPA exposure caused abnormal proliferation of spermatogenic cells (**A**, **B**, **C**, **D**) are representative PCNA immunohistochemical staining images of testis sections from control (coin oil), BPA (0.01 mg/kg), BPA (0.1 mg/kg) and BPA (5 mg/kg) groups, respectively. (**E**) Quantification of PCNA staining as relative percentage to control (as reveled by mean optical density). (**F**) Representative Western blot results from 6 different experiments show protein levels of PCNA in testes of each group with β-actin as a loading reference. (**G**) Ratios of optical density of immunoreactive bands (PCNA vs. β-actin) in testes of each group. Scale bar = 50 μm. Columns, mean; bars, SD (*n* = 6). *p < 0.05, **p < 0.01 compared with control.

Immunoreactivity for PCNA was observed in the nuclei of spermatogonia and some spermatocytes in control group (Figure 3A). The intensity and distribution pattern of PCNA immunostaining were obviously different in BPA-treated groups. In BPA-treated testes, there were more layers of PCNA positive cells in seminiferous tubules and cells located at the center of many seminiferous tubules were PCNA positive (Figure 3B–3D), which were rarely seen in the control testes (Figure 3A). The PCNA positive staining (revealed by mean optical density (MOD)) was significantly increased in the BPA-treated groups (*p* < 0.05, Figure 3E). In addition to immunohistochemical staining, western blotting also confirmed that the expression of PCNA was markedly increased in BPA treatment (*p* < 0.01, Figure 3F, 3G). Together, these findings indicate that BPA exposure caused abnormal proliferation of spermatogenic cells in the developing testes.

### BPA exposure inhibited *BOULE* expression

**Figure 4:**
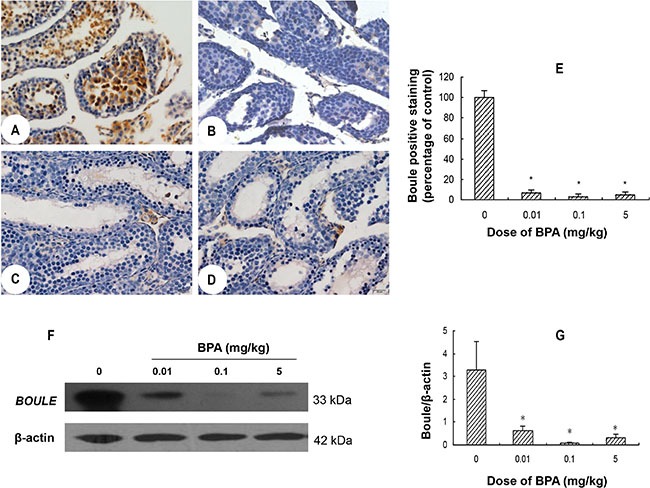
BPA exposure inhibited the expression of *BOULE* in testes (**A**, **B**, **C**, **D**) are representative *BOULE* immunohistochemical staining images of testis sections from control (coin oil), BPA (0.01 mg/kg), BPA (0.1 mg/kg) and BPA (5 mg/kg) groups, respectively. (**E**) Quantification of BOULE positive cells in BPA-treated groups is expressed as relative percentage to control (relative percentage of mean optical density). (**F**) Representative Western blot results from 6 different experiments show protein levels of *BOULE* in testes of each group with β-actin as a loading reference. (**G**) Ratios of optical density of immunoreactive bands (*BOULE* vs. β-actin). Scale bar = 50 μm. Columns, mean; bars, SD (*n* = 6). *p < 0.05 compared with control.

Immunohistochemistry results showed that in control group, abundant *BOULE* protein was present in the cytoplasm of primary spermatocytes through round spermatids (Figure 4A). While in BPA-treated groups, no expression was found in the abovementioned cells (Figure 4B–4D). Western blot results showed a clear band of 33 kDa corresponding to endogenous *BOULE* protein in control group, but such expression was markedly decreased in all BPA-treated groups (*p* < 0.05, Figure 4F, 4G).

### BPA exposure increased testicular ERα and ERβ expression

**Figure 5:**
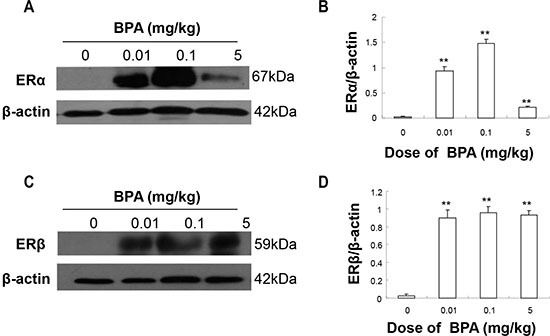
BPA exposure increased the protein levels of ERα and ERβ in testes The protein levels of ERα (**A**) and ERβ (**C**) with β-actin as a loading reference. Representative results from 6 different Western blot experiments. (**B**) and (**D**) show the ratios of optical density of the immunoreactive bands (ERα vs. β-actin for B and ERβ vs. β-actin for D). Columns, mean; bars, SD (*n* = 6). **p < 0.01 compared with control.

We also performed Western blot analysis to determine the expression levels of the estrogen receptors ERα/β in the testes. In the testes of control group (PND 22), minimal immunoreactive bands of ERα and ERβ were detected, but significant increases were observed in ERα and ERβ expression in the BPA-treated groups (*p* < 0.01, Figure 5). The increases in ERα and ERβ expression levels were not in a dose-dependent manner and the highest expression was observed in the middle dose group (0.1 mg/kg) (Figure 5).

## DISCUSSION

Neonatal stage is a very critical development period that is susceptible to chemical exposure [8, 25–27]. Although there are models to study the impacts of chemicals in adult animals, it is not easy to identify the effects of neonatal exposure due to the difficulty of oral administration. Studies have found the enzyme that conjugates BPA (UDP-glucuronosyl-transferase) is expressed at low levels in neonates, and no differences in pharmacokinetics between oral and non-oral dosing was reported [28, 29]. Therefore, in the present study, BPA was administered to male neonatal mice via subcutaneous injection to investigate whether neonatal BPA exposure impaired the first wave of spermatogenesis. The first wave of spermatogenesis begins after birth and is highly synchronized, therefore, neonatal exposure provides a convincing model for assessing the impact of chemicals on meiotic progression [30]. Using this neonatal exposure model, we found neonatal exposure of BPA caused meiotic arrest at the stage of spermatocyte during the first wave of spermatogenesis.

To investigate the molecular mechanisms underpinning the meiotic arrest in spermatogenesis induced by BPA, we assessed the expression levels of *BOULE* (a key regulator of meiosis) and PCNA (a marker of cell proliferation) in the testes after BPA exposure. Interestingly, we found BPA significantly inhibited the expression of *BOULE* and increased the expression of PCNA. *Boule* is conserved across species, exclusively expressed in germ cells, and seems to be essential for completion of spermatogenesis. Lack of *BOULE* expression was correlated with meiosis arrest [16, 17, 31]. *Boule* ortholog was demonstrated to be important for progression beyond pachynema of prophase I of meiosis in *Drosophila* testes [15]. While VanGompel and Xu reported that spermatogenic arrest occurred during the round spermatid stage, prior to elongation in *Boule* knockout mice [18].

Previous studies found that estrogen receptor antagonist blocked BPA-induced meiotic disruption, which implied that estrogen receptor maybe involved in mediating meiotic arrest [32]. Another study showed that BPA exposure (0, 20 and 40 μg/kg daily from PND 3 to PND 35) significantly increased ERα, not ERβ, expression in testis of mouse [33]. Neonatal mice treated with 20 μg of BPA for 5 days since birth also resulted in an increase in the number of ERα-positive cells in the epithelium of the vas deferens at PND18 after birth, but no significant change of ERα expression was found in a higher dose (50 μg BPA) group [34]. In line with the above studies, the induction we observed was not linear, as the highest induction for ERα was achieved by the middle dose (0.1 mg/kg) and all three doses caused similar induction for ERβ. In addition to ERα and ERβ, the most desquamated cells caused by BPA exposure, the highest expression of PCNA, and the least expression of *BOULE* were all observed in the middle dose group (0.1 mg/kg), which further indicates the existence of a nonmonotonic dose-response relationship of BPA. Our findings are consistent with several *in vivo* and *in vitro* studies, which reported the dose-response curve of BPA is indeed an inverted-U-shaped curve [35, 36].

ERα and ERβ participate in the control of proliferation and/or apoptosis of germ cells [37, 38]. In the present study, the elevated expression levels of ERα and ERβ likely contributed to the abnormal proliferation of germ cells as revealed by significantly increased PCNA expression. Zhang *et al.* found a marked increase in the number of germ cells entering meiosis in mice daily treated with 40 μg/kg BPA for 3 weeks [33]. An *in vitro* study also demonstrated that BPA induced spermatogonial cell (GC)-1 cell proliferation [35], whereas the BPA exposure was only maintained 12 h in this experiment and the fate of the proliferating cells weren't predicted. It could be that germ cells were pushed into a state of abnormal proliferation, which will then trigger apoptosis. In the present study, we observed associated abnormal proliferation and elevated apoptosis inside seminiferous tubules of BPA-treated testes. Similarly, Zhang *et al.* also found a sharp decrease in the number of germ cells entering meiosis after treatment with 40 μg/kg BPA for 5 weeks [33].

The present study indicated that neonatal BPA exposure induced meiotic arrest and increased apoptosis of spermatogenic cells during the first wave of spermatogenesis. The lack of *BOULE* expression is likely to contribute to the observed meiotic arrest. The up-regulation of ERα, ERβ and PCNA may be involved in the abnormal cell division induced by BPA. In addition, the present study suggests that neonatal exposure could be considered as a good model to assess the chemical impact on spermatogenesis.

## MATERIALS AND METHODS

### Animals and BPA treatment

SPF *mus muculus* (ICR strain, 10 weeks of age) were purchased from the Shanghai Laboratory Animal Co., Ltd. (SLAC, China), housed in polypropylene cages and acclimatized to an environmentally controlled room (room temperature 24 ± 2°C, relative humidity 40–50%, frequent ventilation and 12 h light-dark cycle). A commercial pellet diet (BPA-free, SLAC, China) and drinking water (in glass bottles) were available ad libitum. The experimental protocol was in accordance with the Guide for the Care and Use of Laboratory Animals and approved by the Institutional Animal Care and Use Committee of Weifang Medical University.

**Figure 6:**
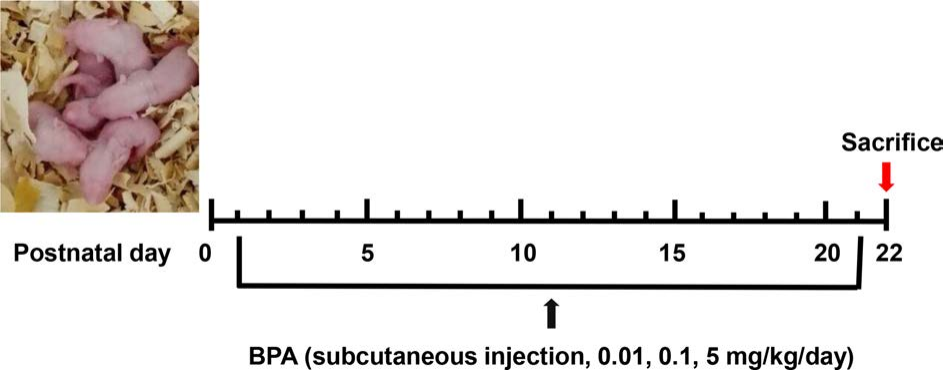
Schematic of animal treatment procedure The day of parturition was designated as PND 0, and BPA treatment started on PND 1. BPA was administered subcutaneously at doses of 0.01, 0.1, and 5 mg/kg/day through PND 21. On PND 22, animals were sacrificed and tissues collected.

After delivery, male pups were randomly divided into four groups (*n* = 16–20 per group) and subcutaneously injected daily with BPA (0.01, 0.1, 5 mg/kg, 4 μl/g, purchased from Sigma-Aldrich, dissolved in absolute ethanol and then diluted in corn oil) or with vehicle (control group) from PND 1 to 21, with the day of birth designated as PND 0, as illustrated in Figure 6. Within 24 h of delivery, female pups were removed and each dam was left with 4 to 5 male pups. At PND 22, male offspring were euthanized by CO_2_ inhalation and testes collected, either stored at −80°C for further molecular analyses or immediately fixed in 4% paraformaldehyde, embedded in paraffin, and sectioned for hematoxylin and eosin (H & E) or immunohistochemical staining.

### Morphological analysis of testes using H & E staining

Paraffin-embedded testes were sectioned on a microtome into 5 μm-thick sections and stained with hematoxylin and eosin for histological examination of testis morphology. Staining was visualized under a light microscope and images were captured with a camera (Leica DFC420, Germany).

### TUNEL assay

Paraffin-embedded testis sections were stained using the *in situ* apoptosis detection TUNEL kit (Roche, Switzerland) following manufacturer's instructions. Sections were then counter stained with hematoxylin. Apoptosis (positive TUNEL staining) of testicular cells was assessed with randomly chosen seminiferous tubules (100 tubules per mouse).

### PCNA and *BOULE* expression by immunohistochemistry

Sections (5 μm-thick) were hydrated and microwaved in 10 mM citrate buffer (pH 6) for antigen retrieval. Endogenous peroxidase and nonspecific binding were then blocked with 0.3% H_2_O_2_ in methanol and 1.5% normal goat serum in 0.01 M PBS, respectively. Sections were incubated overnight in a humidified chamber at 4°C with mouse anti-PCNA polyclonal antibody (Santa Cruz Biotechnology, Inc.) or rabbit anti-*BOULE* polyclonal antibody (abcam). After washing, sections were incubated with rabbit anti-mouse IgG-HRP or goat anti-rabbit IgG-HRP (Santa Cruz Biotechnology, Inc.). Then the sections were washed and incubated with streptavidin-peroxidase complex reagent (Santa Cruz Biotechnology, Inc.) for 15 min. This step was followed by another wash with PBS and subsequent reaction with 3, 3′-diaminobenzidine (DAB) solution (Santa Cruz Biotechnology, Inc.) for color development in the dark. Negative controls were processed simultaneously by omitting the primary antibody. The Integral Optical Density (IOD) was quantified with Image-Pro Plus 6.0 (IPP 6.0) software. The index of MOD (IOD/area) was used to reflect the expression of PCNA and *BOULE*. For each group, 20-30 randomly chosen fields were analyzed.

### Western blot analysis

Frozen tissues were homogenized in PER-Tissue Protein Extraction Buffer (1:20 w:v) (Pierce, Rockford, IL) containing 100 μM phenylmethylsulfonyl fluoride. The homogenates were centrifuged at 20,000 g for 10 min at 4°C. Supernatants (cytosols) were aliquoted and kept at −80°C freezer until use. Protein concentrations were determined using the bicinchoninic acid (BCA) protein assay. Equal amount of proteins were loaded onto each lane of a 12% polyacrylamide gel. The proteins were separated and electroblotted onto a polyvinyl difluoride (PVDF) membrane (Bio-Rad) at 25 V for 60 min using a Trans-Blot (TE77, GE) semidry transfer apparatus. Membranes were blocked in 5% nonfat dry milk for 1 h at room temperature and then incubated with mouse anti-PCNA polyclonal antibody, rabbit anti-*BOULE* polyclonal antibody, rabbit anti-ERα polyclonal antibody, rabbit anti-ERβ polyclonal antibody or mouse anti-β-actin polyclonal antibody (1:1000; Santa Cruz Biotechnology, Inc.) overnight at 4°C. After incubation with horseradish peroxidase-conjugated secondary antibody (Santa Cruz Biotechnology, Inc.), immunoblots were visualized on film using SuperSignal Chemiluminescence Substrate (Pierce Biotechnology).

### Statistical analysis

The data were expressed as the mean ± SD. One-way ANOVA and the Least-Significant Difference test were used to determine differences among different groups. The analyses were performed using Statistical Product and Service Solutions (SPSS, version 11.5). *P* < 0.05 was considered statistically significant.
